# Association Between Neutrophil-to-High-Density Lipoprotein-Cholesterol Ratio and Coronary Artery Calcium: A Cross-Sectional Study

**DOI:** 10.3390/biomedicines14071503

**Published:** 2026-07-02

**Authors:** Yanmiao Liu, Yanqiu Yu, Xinyue Fan, Yangwei Cai, Wenjie Tian

**Affiliations:** 1 Department of Cardiology, Sichuan Provincial People’s Hospital, School of Medicine, University of Electronic Science and Technology of China, Chengdu 610054, China; 202522130307@std.uestc.edu.cn (Y.L.); 202422130305@std.uestc.edu.cn (X.F.); 2 Medical Information Center, Sichuan Provincial People’s Hospital, School of Medicine, University of Electronic Science and Technology of China, Chengdu 610054, China; yuyanqiu@med.uestc.edu.cn

**Keywords:** coronary artery calcium, neutrophil-to-high-density lipoprotein-cholesterol ratio, risk factor, prevention

## Abstract

**Background**: Inflammation and lipid metabolism play critical roles in coronary artery calcium (CAC) progression. This study aimed to investigate the relationship between neutrophil-to-high-density lipoprotein-cholesterol ratio (NHR) and CAC score. **Methods**: This cross-sectional study included 2193 eligible participants from Sichuan Provincial People’s Hospital between November 2015 and July 2025. The correlation between NHR and CAC score was evaluated using multivariable logistic and multinomial logistic regression models. Restricted cubic splines (RCS) were employed to assess potential nonlinear relationships. Sensitivity analyses and subgroup analyses were performed to test the robustness of the findings. **Results**: Among 2193 eligible participants, 64.89% had detectable CAC. Higher NHR levels were significantly associated with increased CAC prevalence. After adjustment for multiple confounders, each 1-unit increase in NHR was associated with 9.0% higher odds of CAC (odds ratio (OR): 1.09, [95% confidence interval (CI) 1.03–1.16], *p* = 0.002). Compared with the lowest NHR quartile, the highest quartile was associated with modestly higher odds of CAC (OR: 1.43 [95% CI 1.05–1.95], *p* = 0.022). In multinomial analyses, NHR was modestly but significantly associated with CAC across mild, moderate, and severe calcification stages. The RCS analysis showed a linear relationship between NHR and CAC. Subgroup analyses and sensitivity analyses confirmed the robustness of the findings. **Conclusions**: Elevated NHR was associated with an increased likelihood of CAC. As a simple and readily available marker, NHR may reflect inflammatory and lipid metabolic status related to subclinical atherosclerosis, although its clinical utility requires further confirmation.

## 1. Introduction

Cardiovascular diseases (CVDs) remain the leading cause of global mortality, with atherosclerosis serving as the foundational pathological substrate for myocardial infarction, stroke, and heart failure [1,2]. Within this continuum, coronary artery calcium (CAC) has emerged as the most robust noninvasive biomarker of cumulative atherosclerotic burden [3,4]. Unlike conventional risk factors or even coronary stenosis severity, CAC reflects histologically advanced, mineralized plaque that is strongly predictive of future cardiovascular events—independently of traditional risk scores, with superior discrimination and reclassification capacity [5,6]. Indeed, CAC scoring is now recognized not merely as an imaging test but as a quantitative measure of disease history—a fossil record of lifelong exposure to atherogenic insults [5,6].

Concurrently, inflammation has been established as a central driver—not just a bystander—in atherosclerosis progression and plaque destabilization. Neutrophils, once considered short-lived first responders, are now understood to orchestrate key pathogenic processes: promoting endothelial dysfunction, enhancing oxidative stress, releasing neutrophil extracellular traps (NETs) that activate macrophages and smooth muscle cells, and directly contributing to fibrous cap thinning and rupture [7,8]. In parallel, high-density lipoprotein cholesterol (HDL-C) functions far beyond simple cholesterol transport; its anti-inflammatory, antioxidant, and reverse cholesterol transport capacities are essential for maintaining vascular homeostasis [9]. Critically, however, HDL-C function—not just concentration—is compromised in chronic inflammatory states, rendering HDL-C levels alone an insufficient metric of protection [10]. The neutrophil-to-high-density lipoprotein-cholesterol ratio (NHR) therefore represents a biologically coherent, integrative index: it quantifies the dynamic imbalance between a pro-atherogenic effector cell and an anti-atherogenic lipoprotein particle [11,12]. Empirical evidence confirms NHR’s prognostic power across diverse CVD phenotypes—including incident coronary artery disease (CAD), all-cause mortality, no-reflow phenomenon post-intervention, and cardiovascular–kidney–metabolic syndrome—often outperforming isolated neutrophil count or HDL-C alone [7,13,14]. However, despite NHR’s ability to integrate systemic inflammatory burden with reduced HDL-C levels, its association with CAC burden—a structural marker of advanced and cumulative atherosclerosis—remains insufficiently characterized in clinical cohorts.

Therefore, this study aimed to investigate the association between NHR and CAC in a large hospital-based cross-sectional study, providing new epidemiological insights into the role of inflammation and lipid metabolism in coronary atherosclerosis.

## 2. Materials and Methods

### 2.1. Study Population

**Figure 1:**
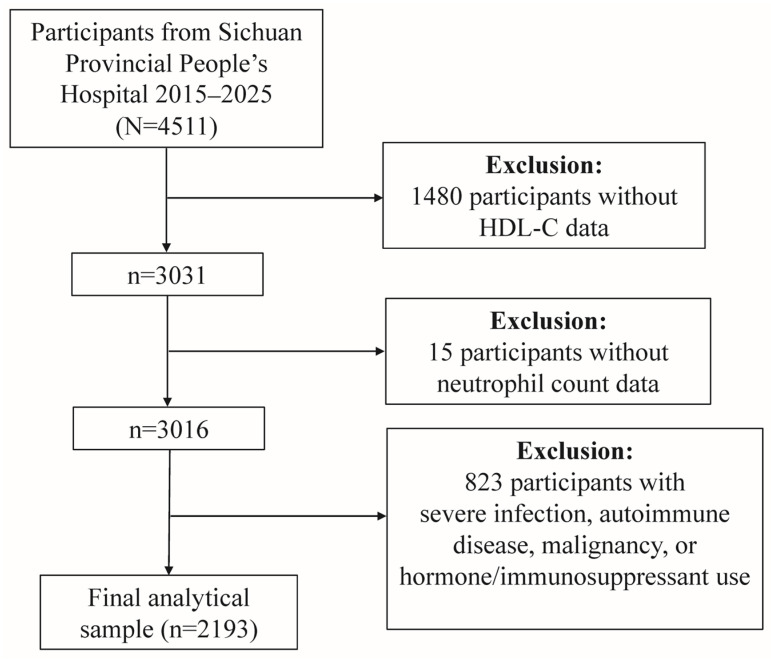
Flowchart for selecting the participants for analysis.

The study employed a cross-sectional research design and included 4511 inpatients who underwent coronary computed tomography angiography (CCTA) at Sichuan Provincial People’s Hospital between November 2015 and July 2025. Of the 4511 participants, 2318 were excluded for any of the following reasons: 1480 with missing HDL-C data, 15 with missing neutrophil count data, and 823 with severe infection (fungal infection, candidiasis, sepsis, severe pneumonia, or immunosuppressive host pneumonia), autoimmune diseases (systemic lupus erythematosus, rheumatoid arthritis, systemic osteoarthritis, or connective tissue disease), malignancy, or use of hormones or immunosuppressants. The total number of eligible subjects for the study was 2193 (Figure 1). This study was approved by the Institutional Review Board of Sichuan Academy of Medical Sciences & Sichuan Provincial People’s Hospital, which exempted the requirement for informed consent as we only accessed de-identified data retrospectively.

### 2.2. Assessment of NHR and CAC

Peripheral blood neutrophil counts were measured using a Mindray BC-6800 Plus analyzer (Mindray, Shenzhen, China) based on flow cytometry and impedance principles as part of routine complete blood count testing. HDL-C was determined by polyanion polymer/detergent assay [15]. NHR was calculated as the ratio of absolute neutrophil count (1000 cells/μL) to HDL-C concentration (mmol/L). CAC assessment was performed using a Siemens SOMATOM Force dual-source CT scanner (Siemens Healthineers, Forchheim, Germany). Prospectively electrocardiography-triggered non-contrast cardiac CT scans were acquired for CAC quantification, with automatic tube voltage selection ranging from 70 to 120 kV and a slice thickness of 0.6 mm. CAC scores were calculated using the Agatston method [16] with dedicated Siemens post-processing software (syngo.via VB40). Calcified lesions were identified using a threshold of ≥130 Hounsfield units, and the total CAC score was calculated by summing lesion-specific scores across the major coronary arteries, including the left main, left anterior descending, left circumflex, and right coronary arteries. Two blinded radiologists independently reviewed CAC scoring, and discrepancies were resolved by consensus or adjudicated by a senior third reader when necessary. The presence of CAC was defined as a CAC score > 0.

### 2.3. Covariates

This study included sociodemographic and health-related covariates, including sex, age, ethnicity, smoking status, alcohol consumption status, body mass index (BMI), and comorbid conditions. Ethnicity was classified as Han Chinese or ethnic minority groups, including Tibetan, Hui, Tujia, Zhuang, Miao, and Yi. Blood biomarkers included estimated glomerular filtration rate (eGFR) and lipoprotein(a). BMI was categorized according to established clinical cutoffs (<24 kg/m^2^, 24–28 kg/m^2^, ≥28 kg/m^2^) [17]. Smoking and drinking status were dichotomized as current versus non-current. The presence of hypertension, diabetes, and CAD, as well as the use of antihypertensive, antidiabetic, and lipid-lowering medications, was determined based on self-reported physician diagnoses and prescription medication use.

### 2.4. Statistical Analysis

Baseline characteristics of the study population were summarized using descriptive statistics. Continuous variables were expressed as means ± standard deviations (SD), and categorical variables were presented as percentages. Group differences were assessed using analysis of variance for continuous variables and chi-square tests for categorical variables. Missing data were handled using multiple imputation by chained equations with the ‘*mice*’ package in R, under the missing-at-random assumption. We generated five imputed datasets, with five iterations for each imputation. Predictive mean matching was used for continuous variables, whereas logistic regression was used for binary variables. The imputation model included NHR, CAC outcomes, and all covariates used in the multivariable models. Convergence was assessed using trace plots of means and SDs. The primary analyses, including multivariable logistic regression and multinomial logistic regression, were conducted across the five imputed datasets, and pooled estimates were obtained using Rubin’s rules [18]. The proportions of missing data and baseline characteristics of participants according to NHR quartiles are presented in Table 1.

Multivariable binary logistic regression models were used to evaluate the association between NHR and the presence of CAC, defined as a CAC score > 0 versus a CAC score of 0. Because CAC reflects an ordered spectrum of subclinical atherosclerosis severity, ordinal logistic regression was initially considered. However, given the violation of the proportional odds assumption, multinomial logistic regression analyses were further performed to examine the association between NHR and CAC severity categories. CAC severity was categorized as 0, 1–100, 101–400, and >400, with CAC score 0 used as the reference category. Sequential models with increasing degrees of adjustment were constructed. Model 1 was unadjusted; Model 2 was adjusted for age and sex; and Model 3 was further adjusted for ethnicity, BMI, eGFR, lipoprotein(a), smoking status, drinking status, hypertension, diabetes, and the use of antihypertensive, antidiabetic, and lipid-lowering medications. NHR was modeled both as a continuous variable and as a categorical variable according to quartiles. Tests for trend were performed by assigning the median value of each NHR quartile and modeling this variable as a continuous term in the corresponding multivariable logistic regression models.

Restricted cubic spline (RCS) regression with three knots was used to explore potential nonlinear associations between NHR and CAC [19]. The knots were selected based on the Akaike information criterion, and the reference value was set at the median NHR, corresponding to an odds ratio (OR) of 1.0. Overall associations and nonlinearity were assessed using likelihood ratio tests. In addition, Spearman correlations of NHR, neutrophil count, and HDL-C with log-transformed CAC score [log(CAC + 1)] were assessed. Scatter plots with locally estimated scatterplot smoothing curves were generated and are presented in Supplemental Figure S1.

Subgroup analyses were conducted stratified by age, sex, ethnicity, smoking status, drinking status, and the use of antihypertensive, antidiabetic, and lipid-lowering medications. Potential effect modification was assessed by testing multiplicative interaction terms between NHR and each subgroup variable using likelihood ratio tests, comparing multivariable logistic regression models with and without the interaction terms. To evaluate the robustness of the primary findings, several sensitivity analyses were performed. First, missing-indicator methods were applied as an alternative approach to handling missing covariate data. Second, complete-case analyses were conducted by excluding participants with missing covariate data. Finally, to account for potential confounding by pre-existing CVD, additional models were fitted with further adjustment for prevalent CAD.

All statistical analyses were carried out using R software (version 4.5.3, http://www.r-project.org/ (accessed on 27 March 2026)), with statistical significance defined as a two-sided *p* value < 0.05.

## 3. Results

### 3.1. Baseline Characteristics According to NHR Quartiles

Among the study participants, the mean age was 67.14 ± 12.27 years, and 1311 (59.78%) were men. Overall, 1423 (64.89%) participants had detectable CAC, defined as a CAC score > 0 (Table 1). Participants were categorized into four groups according to baseline NHR quartiles (Table 1). Higher NHR quartiles were associated with a progressively higher CAC incidence (*p* = 0.006) and higher CAC scores (*p* = 0.002). Although Spearman analyses revealed only weak correlations between log(CAC + 1) and NHR (rho = 0.096, *p* < 0.001) or neutrophil count alone (rho = 0.091, *p* < 0.001), HDL-C showed no significant correlation (Supplemental Figure S1).

Participants in higher NHR quartiles were generally younger and more likely to be male and current smokers. BMI increased across ascending NHR quartiles. In addition, higher NHR levels were associated with a higher prevalence of comorbid conditions, including hypertension, diabetes, and CAD, as well as lower eGFR, reflecting impaired renal function. Consistently, participants with higher NHR levels were more likely to use related medications, including antihypertensive, antidiabetic, and lipid-lowering therapies (all *p* < 0.001).

### 3.2. Association Between NHR and CAC

**Figure 2:**
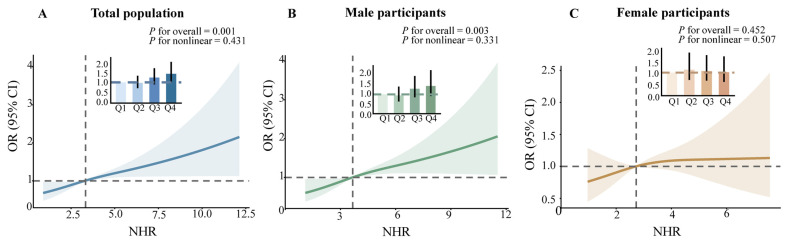
Restricted cubic spline (RCS) curves demonstrate associations of baseline neutrophil-to-high-density lipoprotein-cholesterol ratio (NHR) with coronary artery calcium in: (**A**) the total population, (**B**) male participants, and (**C**) female participants. The colored lines represent the fitted RCS curves showing adjusted odds ratios (ORs); the shaded areas represent the 95% confidence intervals (CIs); the dashed horizontal lines indicate OR = 1.0 (the null value of no association); and the dashed vertical lines indicate the reference NHR value where the OR is set to 1.0. Bar plots in insets show ORs (95% CIs) by NHR quartiles, with quartile 1 (Q1, the lowest NHR) as the reference group, and quartiles 2–4 (Q2–Q4) representing progressively higher NHR levels.

When NHR was analyzed as a continuous variable in the binary logistic regression model, each 1-unit increase in NHR was associated with 9.0% higher odds of CAC after adjustment for potential confounders (OR, 1.09; 95% confidence interval (CI), 1.03–1.16; *p* = 0.002). In the quartile-based analysis, participants in the highest NHR quartile had modestly but significantly higher odds of CAC than those in the lowest quartile (OR, 1.43; 95% CI, 1.05–1.95; *p* = 0.022), suggesting that elevated NHR was associated with a greater likelihood of detectable CAC. The RCS analysis further revealed a significant positive linear association between NHR and the presence of CAC in the overall population (*p* for overall = 0.001 and *p* for nonlinear = 0.431, Figure 2A). In multinomial logistic regression analyses with CAC stratified by severity, NHR remained modestly but significantly associated with all CAC severity categories after full adjustment (OR, 1.08; 95% CI, 1.02–1.15 for CAC score 1–100; OR, 1.11; 95% CI, 1.04–1.19 for CAC score 101–400; and OR, 1.09; 95% CI, 1.01–1.17 for CAC score >400). Quartile-based analyses showed that significant associations were largely confined to participants in the highest NHR quartile (Supplemental Table S1).

Sex-stratified analyses showed that the positive association between NHR and CAC appeared more evident in men than in women (Table 2). Among men, NHR was significantly associated with CAC when modeled as a continuous variable, albeit with a modest effect size (OR, 1.11; 95% CI, 1.04–1.19; *p* = 0.003). Participants in the highest NHR quartile also had modestly but significantly higher odds of CAC than those in the lowest quartile (OR, 1.69; 95% CI, 1.12–2.55; *p* = 0.013). In contrast, among women, NHR was not significantly associated with CAC when analyzed as a continuous variable in the fully adjusted model (OR, 1.05; 95% CI, 0.94–1.17; *p* = 0.423), and no significant trend was observed across quartiles (*p* for trend = 0.684). Consistently, sex-stratified RCS analysis showed a predominantly linear association between NHR and the presence of CAC in men (Figure 2B), whereas the association was not statistically significant in women (Figure 2C).

### 3.3. Subgroup and Sensitivity Analyses

**Figure 3:**
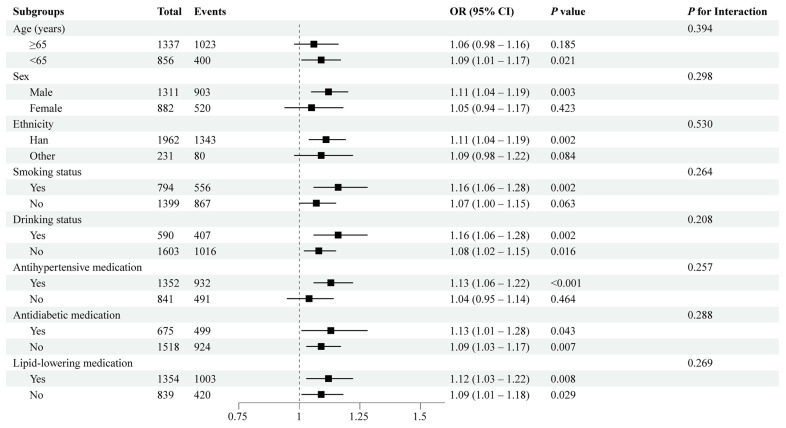
Subgroup analysis of the association between baseline neutrophil-to-high-density lipoprotein-cholesterol ratio and coronary artery calcium. Logistic regression was performed after adjustment for age, sex, ethnicity, body mass index categories, estimated glomerular filtration rate, lipoprotein(a), smoking status, drinking status, antihypertensive medication use, antidiabetic medication use, lipid-lowering medication use, hypertension, and diabetes, except for the stratification variable itself.

To evaluate the consistency of the association between NHR and CAC, subgroup analyses were conducted using multivariable logistic regression stratified by age, sex, ethnicity, smoking status, drinking status, and use of antihypertensive, antidiabetic, or lipid-lowering medications (Figure 3). The positive association between NHR and CAC was generally consistent across most prespecified subgroups. Although the association appeared more pronounced among participants aged < 65 years, individuals of Han ethnicity, smokers, and users of antihypertensive medications, no significant interactions were observed across subgroup strata (all *p* for interaction > 0.05).

Sensitivity analyses yielded concordant results: the association remained statistically significant in multivariable logistic regression based on missing indicator method or complete-case analyses (Supplemental Tables S2 and S3) and after further adjustment for prevalent CAD (Supplemental Table S4).

## 4. Discussion

Our study provides novel epidemiological evidence of a statistically significant and largely linear association between NHR and CAC in a hospital-based cohort of inpatients undergoing clinically indicated CCTA. After multivariable adjustment for age, sex, hypertension, diabetes, smoking and drinking status, BMI, eGFR, lipoprotein(a), and relevant medication use, elevated NHR remained independently associated with higher CAC prevalence. Notably, this association was largely linear across the observed NHR range, with gradual increases in the odds of CAC as NHR increased. Furthermore, NHR was modestly but significantly associated with CAC across different calcification severity categories. The strongest linear trend was observed for severe calcification (CAC score > 400; *p* for trend = 0.010), although the effect sizes were modest, and significant associations were mainly concentrated among participants in the highest NHR quartile. A series of subgroup and sensitivity analyses confirmed the robustness of our findings. These results suggest that NHR may be associated with subclinical atherosclerotic burden, potentially reflecting concurrent immunometabolic dysregulation rather than systemic inflammation alone.

Atherosclerosis and vascular calcification are closely linked to chronic inflammation and dysregulated lipid metabolism. As a simple and readily available biomarker, NHR reflects the interplay between systemic inflammation and lipid metabolism—two central mechanisms implicated in atherosclerosis and vascular calcification [20,21]. Accumulating evidence has demonstrated significant associations between NHR and atherosclerotic cardiovascular disease. Recent studies have shown that NHR is associated with the progression and severity of acute coronary syndrome in individuals with type 2 diabetes [22], correlates with the occurrence of CAD [23], and reflects the severity of coronary stenosis [11]. In addition, a previous study of US adults demonstrated an association between NHR and abdominal aortic calcification [24]. Extending this body of evidence, our cross-sectional analysis further demonstrates an association between NHR and CAC, a quantifiable marker of subclinical and structural atherosclerosis. By linking NHR to CAC burden, our findings complement prior studies and suggest that NHR may be associated with different stages along the continuum of CAD progression.

The biological mechanisms underlying the association between NHR and CAC are not yet fully elucidated; however, several plausible pathways may be involved. First, neutrophil-driven osteogenic transdifferentiation may play a central role. Activated neutrophils infiltrate the arterial intima and release NETs, which contain histones, myeloperoxidase, and neutrophil elastase [25]. These components directly stimulate vascular smooth muscle cells to undergo phenotypic switching toward osteoblast-like cells, upregulating RUNX2, BMP2, and alkaline phosphatase—key drivers of hydroxyapatite deposition [26,27]. Animal models also confirm that NET deposition colocalizes with calcified lesions and accelerates vascular calcification in vivo [28]. Second, reduced HDL-C and impaired HDL-mediated vascular protection may further contribute to this process. HDL-C exerts well-established anti-atherogenic and anti-inflammatory effects, including promotion of reverse cholesterol transport, inhibition of low-density lipoprotein oxidation, and preservation of endothelial function [9,29]. Consequently, the coexistence of elevated neutrophil counts and reduced HDL-C levels—captured by an increased NHR—may be associated with a synergistic state of heightened inflammatory burden and impaired lipid-mediated vascular protection. This composite index may therefore be more closely associated with the pathological milieu that accompanies CAC than either neutrophil count or HDL-C alone.

In addition, the association between NHR and CAC appeared to be more evident in men than in women, although interaction testing did not indicate a statistically significant effect modification by sex. Sex differences in CAC have been well documented, with prior studies showing that men tend to have a higher burden and earlier onset of CAC than women [30,31]. This sex-related pattern may partly explain the more pronounced association observed between NHR and CAC among men. Taken together, these findings suggest that NHR may be more closely associated with CAC burden in men, although further studies are warranted to determine whether NHR has potential value in sex-specific CAC assessment.

This study has several notable strengths. It focuses on subclinical CAC, the earliest detectable morphological manifestation of coronary atherosclerosis, employs the validated Agatston scoring method for CAC quantification, and incorporates extensive sensitivity analyses that consistently support the robustness of the findings across multiple modeling strategies. Several limitations should also be acknowledged. First, the single-center, retrospective design and the lack of longitudinal CAC measurements preclude causal inference and limit the ability to assess CAC progression over time. Future studies should prioritize multicenter, prospective cohorts with serial CAC assessments and integration of time-varying NHR trajectories to examine whether NHR is associated with calcification progression. Second, although comprehensive covariate adjustment was performed, residual confounding from unmeasured factors—such as dietary patterns, physical activity, psychosocial stress, low-density lipoprotein cholesterol levels, statin use and intensity, and other lipid-lowering therapies—cannot be entirely excluded. Third, the study population comprised inpatients undergoing clinically indicated CCTA and was predominantly of Han Chinese ethnicity, which may limit the generalizability of the findings to other clinical settings, ethnic groups, and geographic populations. Finally, the clinical incremental value of NHR beyond established cardiovascular risk markers remains uncertain, as its ability to improve risk reclassification or decision curve analysis beyond conventional factors was not assessed. Therefore, further prospective studies are warranted to clarify whether NHR is associated with added prognostic information beyond established risk markers.

## 5. Conclusions

In this hospital-based cross-sectional study of inpatients undergoing clinically indicated CCTA, higher NHR was positively associated with a greater likelihood of CAC. These findings suggest that NHR may reflect inflammatory and lipid metabolic profiles associated with subclinical atherosclerosis, although its clinical utility for cardiovascular risk assessment remains to be established. Further longitudinal studies are warranted to determine whether NHR predicts CAC progression and improves cardiovascular risk stratification beyond conventional risk factors.

## Figures and Tables

**Table 1:** Characteristics of the study population according to the neutrophil-to-high-density lipoprotein-cholesterol ratio(NHR) categories.

	Total	Quartile 1	Quartile 2	Quartile 3	Quartile 4	*p* Value
	n = 2193	n = 549	n = 548	n = 548	n = 548	
NHR	3.66 (2.19)	1.78 (0.39)	2.81 (0.28)	3.84 (0.35)	6.21 (2.84)	<0.001
Sex						<0.001
Male	1311 (59.78%)	218 (39.71%)	305 (55.66%)	367 (66.97%)	421 (76.82%)	
Female	882 (40.22%)	331 (60.29%)	243 (44.34%)	181 (33.03%)	127 (23.18%)	
Age, years	67.14 (12.27)	69.46 (11.20)	68.08 (11.62)	66.37 (12.00)	64.66 (13.61)	<0.001
Ethnicity						<0.001
Han	1663 (75.83%)	411 (74.86%)	413 (75.36%)	426 (77.74%)	413 (75.36%)	
Other	182 (8.30%)	44 (8.01%)	40 (7.30%)	39 (7.12%)	59 (10.77%)	
Missing	348 (15.87%)	94 (17.12%)	95 (17.34%)	83 (15.15%)	76 (13.87%)	
BMI categories						<0.001
<24 kg/m^2^	984 (44.87%)	312 (56.83%)	226 (41.24%)	226 (41.24%)	220 (40.15%)	
24–28 kg/m^2^	733 (33.42%)	160 (29.14%)	191 (34.85%)	186 (33.94%)	196 (35.77%)	
≥28 kg/m^2^	271 (12.36%)	38 (6.92%)	62 (11.31%)	79 (14.42%)	92 (16.79%)	
Missing	205 (9.35%)	39 (7.10%)	69 (12.59%)	57 (10.40%)	40 (7.30%)	
Smoking status						<0.001
Yes	730 (33.29%)	126 (22.95%)	162 (29.56%)	207 (37.77%)	235 (42.88%)	
No	1324 (60.37%)	395 (71.95%)	349 (63.69%)	300 (54.74%)	280 (51.09%)	
Missing	139 (6.34%)	28 (5.10%)	37 (6.75%)	41 (7.48%)	33 (6.02%)	
Drinking status						0.001
Yes	507 (23.12%)	93 (16.94%)	131 (23.91%)	146 (26.64%)	137 (25.00%)	
No	1485 (67.72%)	414 (75.41%)	369 (67.34%)	350 (63.87%)	352 (64.23%)	
Missing	201 (9.17%)	42 (7.65%)	48 (8.76%)	52 (9.49%)	59 (10.77%)	
Systolic blood pressure, mmHg	131.14 (20.23)	130.83 (19.64)	131.12 (20.56)	131.80 (19.00)	130.83 (21.62)	0.846
Diastolic blood pressure, mmHg	74.82 (14.09)	73.10 (13.72)	73.65 (12.96)	75.85 (13.76)	76.64 (15.46)	<0.001
Hypertension						<0.001
Yes	1131 (51.57%)	236 (42.99%)	290 (52.92%)	294 (53.65%)	311 (56.75%)	
No	1062 (48.43%)	313 (57.01%)	258 (47.08%)	254 (46.35%)	237 (43.25%)	
Diabetes						<0.001
Yes	571 (26.04%)	86 (15.66%)	142 (25.91%)	168 (30.66%)	175 (31.93%)	
No	1622 (73.96%)	463 (84.34%)	406 (74.09%)	380 (69.34%)	373 (68.07%)	
CAD						<0.001
Yes	621 (28.32%)	125 (22.77%)	139 (25.36%)	171 (31.20%)	186 (33.94%)	
No	1572 (71.68%)	424 (77.23%)	409 (74.64%)	377 (68.80%)	362 (66.06%)	
Antihypertensive medication						<0.001
Yes	1352 (61.65%)	287 (52.28%)	332 (60.58%)	359 (65.51%)	374 (68.25%)	
No	841 (38.35%)	262 (47.72%)	216 (39.42%)	189 (34.49%)	174 (31.75%)	
Antidiabetic medication						<0.001
Yes	675 (30.78%)	113 (20.58%)	162 (29.56%)	195 (35.58%)	205 (37.41%)	
No	1518 (69.22%)	436 (79.42%)	386 (70.44%)	353 (64.42%)	343 (62.59%)	
Lipid-lowering medication						<0.001
Yes	1354 (61.74%)	297 (54.10%)	347 (63.32%)	360 (65.69%)	350 (63.87%)	
No	839 (38.26%)	252 (45.90%)	201 (36.68%)	188 (34.31%)	198 (36.13%)	
HDL-C, mmol/L	1.21 (0.37)	1.54 (0.37)	1.29 (0.28)	1.10 (0.24)	0.91 (0.22)	<0.001
eGFR, mL/min/1.73 m^2^	81.18 (21.77)	82.70 (18.85)	82.76 (19.58)	81.13 (20.82)	78.14 (26.68)	0.004
Lipoprotein(a), nmol/L	86.97 (160.99)	76.28 (132.01)	85.50 (164.14)	82.68 (140.99)	103.70 (198.59)	0.075
Neutrophil count, 1000 cells/μL	3.96 (1.32)	2.68 (0.70)	3.60 (0.81)	4.22 (0.92)	5.34 (1.11)	<0.001
Agatston CAC score	234.47 (502.07)	185.36 (423.92)	204.45 (444.52)	263.97 (555.41)	284.21 (563.36)	0.002
CAC > 0, n (%)	1423 (64.89%)	329 (59.93%)	346 (63.14%)	369 (67.34%)	379 (69.16%)	0.006

BMI, body mass index; CAC, coronary artery calcium; CAD, coronary artery disease; HDL-C, high-density lipoprotein cholesterol; eGFR, estimated glomerular filtration rate.

**Table 2:** Association between neutrophil-to-high-density lipoprotein-cholesterol ratio (NHR) and coronary artery calcium (CAC) (n = 2193).

		OR (95% CI), *p* Value		
	Cases/Participants (%)	Crude Model (Model 1)	Partially Adjusted Model (Model 2)	Fully Adjusted Model (Model 3)
**All participants**				
NHR (as continuous variable)	1423/2193 (64.89%)	1.07 (1.02–1.12), 0.006	1.13 (1.07–1.20), <0.001	1.09 (1.03–1.16), 0.002
NHR Quartiles				
Quartile 1 (<2.34)	329/549 (59.93%)	Reference	Reference	Reference
Quartile 2 (2.34–3.28)	346/548 (63.14%)	1.15 (0.90–1.46), 0.275	1.18 (0.90–1.54), 0.222	1.03 (0.78–1.36), 0.849
Quartile 3 (3.28–4.49)	369/548 (67.34%)	1.38 (1.08–1.76), 0.011	1.56 (1.18–2.05), 0.002	1.23 (0.92–1.65), 0.160
Quartile 4 (≥4.49)	379/548 (69.16%)	1.50 (1.17–1.92), 0.001	1.86 (1.40–2.48), <0.001	1.43 (1.05–1.95), 0.022
*p* for trend		<0.001	<0.001	0.059
**Male**				
NHR (as continuous variable)	903/1311 (68.88%)	1.04 (0.99–1.10), 0.179	1.13 (1.06–1.21), <0.001	1.11 (1.04–1.19), 0.003
NHR Quartiles				
Quartile 1 (<2.69)	143/218 (65.60%)	Reference	Reference	Reference
Quartile 2 (2.69–3.66)	200/305 (65.57%)	1.00 (0.69–1.44), 0.996	1.21 (0.82–1.78), 0.334	1.02 (0.68–1.54), 0.914
Quartile 3 (3.66–4.92)	259/367 (70.57%)	1.26 (0.88–1.80), 0.210	1.71 (1.17–2.51), 0.006	1.41 (0.94–2.13), 0.100
Quartile 4 (≥4.92)	301/421 (71.50%)	1.32 (0.92–1.87), 0.125	2.10 (1.43–3.07), <0.001	1.69 (1.12–2.55), 0.013
*p* for trend		0.050	<0.001	0.003
**Female**				
NHR (as continuous variable)	520/882 (58.96%)	1.05 (0.96–1.15), 0.264	1.11 (1.00–1.23), 0.046	1.05 (0.94–1.17), 0.423
NHR Quartiles				
Quartile 1 (<1.97)	186/331 (56.19%)	Reference	Reference	Reference
Quartile 2 (1.97–2.72)	146/243 (60.08%)	1.17 (0.84–1.64), 0.351	1.19 (0.82–1.73), 0.368	1.03 (0.69–1.53), 0.903
Quartile 3 (2.72–3.78)	110/181 (60.77%)	1.21 (0.84–1.75), 0.316	1.43 (0.95–2.17), 0.092	1.13 (0.73–1.76), 0.589
Quartile 4 (≥3.78)	78/127 (61.42%)	1.24 (0.82–1.89), 0.312	1.33 (0.83–2.16), 0.242	1.05 (0.63–1.77), 0.843
*p* for trend		0.232	0.100	0.684

Model 1: unadjusted model. Model 2: adjusted for sex and age. Model 3: model 2 plus ethnicity, body mass index categories, estimated glomerular filtration rate, lipoprotein(a), smoking status, drinking status, antihypertensive medication use, antidiabetic medication use, lipid-lowering medication use, hypertension, and diabetes. CI confidence interval, OR odds ratio.

## Data Availability

The data that support the findings of this study are available on request from the corresponding author (Dr. Yangwei Cai; email: [caiyangwei@med.uestc.edu.cn]). Data are not publicly available due to restrictions imposed by the Ethics Committee of Sichuan Academy of Medical Sciences & Sichuan Provincial People’s Hospital to protect patient privacy and comply with Chinese regulations on personal information protection and medical data management. Requests for data access will be reviewed by the institutional ethics committee and may require additional ethics approval.

## Supplementary Materials

The following supporting information can be downloaded at: https://www.mdpi.com/article/10.3390/biomedicines14071503/s1, Supplemental Figure S1. Spearman correlation of (A) neutrophil-to-high-density lipoprotein-cholesterol ratio (NHR), (B) neutrophil count, and (C) high-density lipoprotein cholesterol (HDL-C) with log-transformed coronary artery calcium (CAC) score [log(CAC + 1)]. Each dot represents an individual participant. The solid line indicates the fitted locally estimated scatterplot smoothing curve, and the shaded area denotes the 95% confidence interval. rho, Spearman rank correlation coefficient. Supplemental Table S1. Association between neutrophil-to-high-density lipoprotein-cholesterol ratio (NHR) and coronary artery calcium (CAC) stages by multinomial logistic regression (n = 2193). Supplemental Table S2. Association between neutrophil-to-high-density lipoprotein-cholesterol ratio (NHR) and coronary artery calcium (CAC) based on missing indicator methods (n = 2193). Supplemental Table S3. Association between neutrophil-to-high-density lipoprotein-cholesterol ratio (NHR) and coronary artery calcium (CAC) in complete-case analyses (n = 1334). Supplemental Table S4. Association between neutrophil-to-high-density lipoprotein-cholesterol ratio (NHR) and coronary artery calcium (CAC) after adding coronary artery disease (CAD) covariate (n = 2193).

## Abbreviations

The following abbreviations are used in this manuscript:

BMI	Body mass index
CAD	Coronary artery disease
CAC	Coronary artery calcium
CCTA	Coronary computed tomography angiography
CI	Confidence interval
CVD	Cardiovascular disease
eGFR	Estimated glomerular filtration rate
HDL-C	High-density lipoprotein cholesterol
NET	Neutrophil extracellular trap
NHR	Neutrophil-to-high-density lipoprotein-cholesterol ratio
OR	Odds ratio
RCS	Restricted cubic spline
SD	Standard deviation
