# Supplementary material for: Association Between Neutrophil-to-High-Density Lipoprotein-Cholesterol Ratio and Coronary Artery Calcium: A Cross-Sectional Study
Source: Biomedicines. 2026 Jul 2;14(7):1503. doi: 10.3390/biomedicines14071503 (PMC13403793; doi:10.3390/biomedicines14071503)
Supplement: Supplementary file 1 [file biomedicines-14-01503-s001.zip › biomedicines-4338842-supplementary.pdf]

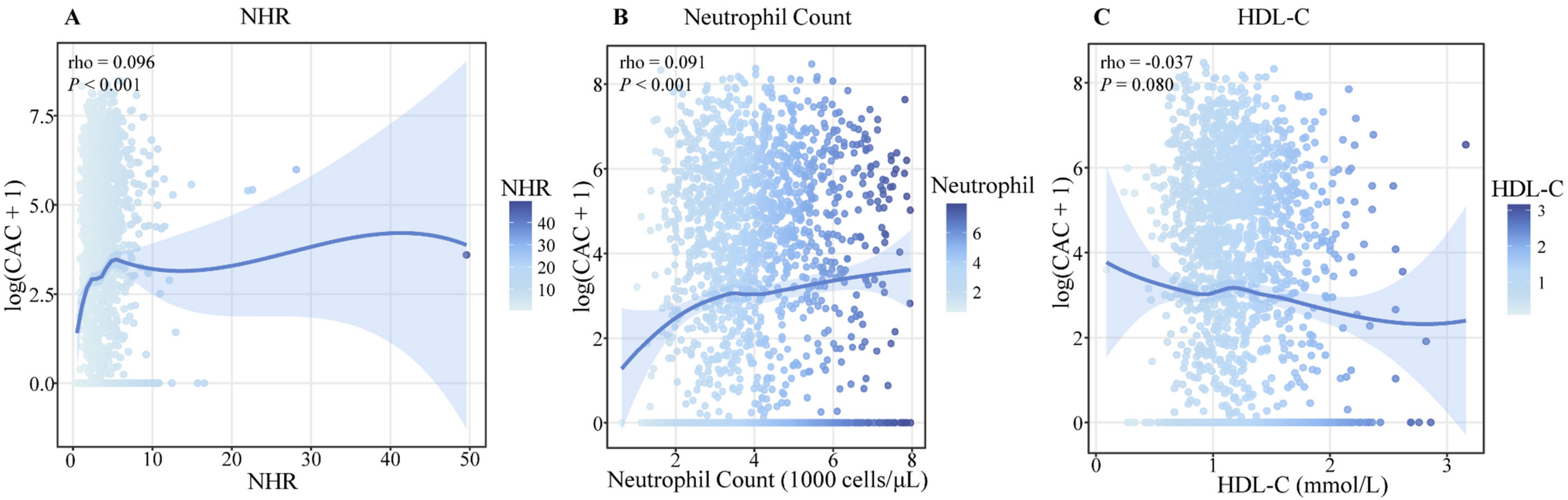

Supplemental Figure S1. Spearman correlation of (A) neutrophil-to-high-density lipoprotein-cholesterol ratio (NHR), (B) neutrophil count, and (C) high-density lipoprotein cholesterol (HDL-C) with log-transformed coronary artery calcium (CAC) score [log(CAC + 1)]. Each dot represents an individual participant. The solid line indicates the fitted locally estimated scatterplot smoothing curve, and the shaded area denotes the 95% confidence interval. rho, Spearman rank correlation coefficient.

Supplemental Table S1. Association between neutrophil-to-high-density lipoprotein-cholesterol ratio (NHR) and coronary artery calcium (CAC) stages by multinomial logistic regression (n= 2193).

	**OR (95% CI), *P* value**		
	**CAC 1–100**	**CAC 101–400**	**CAC >400**
NHR (as continuous variable)			
Model 1	1.06 (1.00–1.12)	1.08 (1.02–1.15)	1.08 (1.02–1.14)
Model 2	1.12 (1.05–1.19)	1.16 (1.08–1.24)	1.14 (1.07–1.22)
Model 3	1.08 (1.02–1.15)	1.11 (1.04–1.19)	1.09 (1.01–1.17)
NHR Quartiles			
Quartile 2 versus Quartile 1			
Model 1	1.25 (0.93–1.67)	1.11 (0.79–1.56)	1.05 (0.73–1.51)
Model 2	1.28 (0.94–1.74)	1.14 (0.80–1.64)	1.05 (0.71–1.54)
Model 3	1.14 (0.82–1.57)	0.93 (0.63–1.36)	0.82 (0.54–1.24)
Quartile 3 versus Quartile 1			
Model 1	1.28 (0.95–1.73)	1.29 (0.92–1.82)	1.61 (1.13–2.28)
Model 2	1.45 (1.05–2.00)	1.50 (1.03–2.17)	1.78 (1.22–2.60)

Model 3	1.20 (0.86–1.69)	1.13 (0.77–1.67)	1.28 (0.85–1.92)
Quartile 4 versus Quartile 1			
Model 1	1.39 (1.03–1.88)	1.45 (1.03–2.04)	1.83 (1.29–2.60)
Model 2	1.73 (1.24–2.42)	1.87 (1.28–2.74)	2.22 (1.51–3.28)
Model 3	1.44 (1.01–2.05)	1.36 (0.90–2.04)	1.54 (1.01–2.36)
P for trend			
Model 1	0.036	0.021	<0.001
Model 2	0.001	<0.001	<0.001
Model 3	0.049	0.094	0.010

Model 1: unadjusted model. Model 2: adjusted for sex and age. Model 3: model 2 plus ethnicity, body mass index categories, estimated glomerular filtration rate, lipoprotein(a), smoking status, drinking status, antihypertensive medication use, antidiabetic medication use, lipid-lowering medication use, hypertension, and diabetes. CI confidence interval, OR odds ratio.

Supplemental Table S2. Association between neutrophil-to-high-density lipoprotein-cholesterol ratio (NHR) and coronary artery calcium (CAC) based on missing indicator methods (n= 2193).

		OR (95% CI), *P* value		
	**Cases/participants (%)**	**Crude model (Model 1)**	**Partially adjusted model (Model 2)**	**Fully adjusted model (Model 3)**
NHR (as continuous variable)	1423/2193 (64.89%)	1.07 (1.02–1.12), 0.006	1.13 (1.07–1.20), <0.001	1.11 (1.05–1.18), <0.001
NHR Quartiles				
Quartile 1 (<2.34)	329/549 (59.93%)	Reference	Reference	Reference
Quartile 2 (2.34–3.28)	346/548 (63.14%)	1.15 (0.90–1.46), 0.274	1.18 (0.91–1.54), 0.222	1.00 (0.74–1.34), 0.984
Quartile 3 (3.28–4.49)	369/548 (67.34%)	1.38 (1.08–1.77), 0.011	1.56 (1.18–2.05), 0.002	1.22 (0.90–1.65), 0.192
Quartile 4 (≥4.49)	379/548 (69.16%)	1.50 (1.17–1.93), 0.001	1.86 (1.40–2.48), <0.001	1.56 (1.13–2.15), 0.007
P for trend		<0.001	<0.001	0.792

Model 1: unadjusted model. Model 2: adjusted for sex and age. Model 3: model 2 plus ethnicity, body mass index categories, estimated glomerular filtration rate, lipoprotein(a), smoking status, drinking status, antihypertensive medication use, antidiabetic medication use, lipid-lowering medication use, hypertension, and diabetes. CI confidence interval, OR odds ratio.

Supplemental Table S3. Association between neutrophil-to-high-density lipoprotein-cholesterol ratio (NHR) and coronary artery calcium (CAC) in complete-case analyses (n= 1334).

	OR (95% CI), *P* value			
	**Cases/participants (%)**	**Crude model (Model 1)**	**Partially adjusted model (Model 2)**	**Fully adjusted model (Model 3)**
NHR (as continuous variable)	867/1334 (64.99%)	1.09 (1.02–1.16), 0.010	1.13 (1.06–1.22), <0.001	1.12 (1.04–1.20), 0.003
NHR Quartiles				
Quartile 1 (<2.30)	206/334 (61.68%)	Reference	Reference	Reference
Quartile 2 (2.30–3.31)	197/333 (59.16%)	0.90 (0.66–1.23), 0.506	0.88 (0.62–1.23), 0.447	0.78 (0.54–1.12), 0.178
Quartile 3 (3.31–4.56)	228/333 (68.47%)	1.35 (0.98 – 1.86), 0.066	1.40 (0.98–2.00), 0.065	1.19 (0.82–1.73), 0.360
Quartile 4 (≥4.56)	236/334 (70.66%)	1.50 (1.08–2.07), 0.014	1.74 (1.20–2.55), 0.004	1.46 (0.98–2.18), 0.065
P for trend		0.002	<0.001	0.960

Model 1: unadjusted model. Model 2: adjusted for sex and age. Model 3: model 2 plus ethnicity, body mass index categories, estimated glomerular filtration rate, lipoprotein(a), smoking status, drinking status, antihypertensive medication use, antidiabetic medication use, lipid-lowering medication use, hypertension, and diabetes. CI confidence interval, OR odds ratio.

Supplemental Table S4. Association between neutrophil-to-high-density lipoprotein-cholesterol ratio (NHR) and coronary artery calcium (CAC) after adding coronary artery disease (CAD) covariate (n = 2193).

		**OR (95% CI), *P* value**		
	**Cases/participants (%)**	**Crude model (Model 1)**	**Partially adjusted model (Model 2)**	**Fully adjusted model (Model 3)**
NHR (as continuous variable)	1423/2193 (64.89%)	1.07 (1.02–1.12), 0.006	1.13 (1.07–1.20), <0.001	1.11 (1.04–1.17), <0.001
NHR Quartiles				
Quartile 1 (<2.34)	329/549 (59.93%)	Reference	Reference	Reference
Quartile 2 (2.34–3.28)	346/548 (63.14%)	1.15 (0.90–1.46), 0.274	1.18 (0.91–1.54), 0.222	0.99 (0.74–1.32), 0.940
Quartile 3 (3.28–4.49)	369/548 (67.34%)	1.38 (1.08–1.77), 0.011	1.56 (1.18–2.05), 0.002	1.21 (0.89–1.63), 0.223
Quartile 4 (≥4.49)	379/548 (69.16%)	1.50 (1.17–1.93), 0.001	1.86 (1.40–2.48), <0.001	1.53 (1.11–2.11), 0.009
P for trend		<0.001	<0.001	0.005

Model 1: unadjusted model. Model 2: adjusted for sex and age. Model 3: model 2 plus ethnicity, body mass index categories, estimated glomerular filtration rate, lipoprotein(a), smoking status, drinking status, antihypertensive medication use, antidiabetic medication use, lipid-lowering medication use, hypertension, diabetes and CAD. CI confidence interval, OR odds ratio.
